# Cross-Frequency Magnetic Modulation of Hippocampal Synaptic Plasticity: From Cellular Mechanisms to System-Level Adaptation

**DOI:** 10.3390/brainsci16080795

**Published:** 2026-07-28

**Authors:** Shufang Deng, Lei Tian, Jianpeng Song, Yameng Zhou, Yongxiang Gao, Shuai Cao

**Affiliations:** School of Life Sciences, Tiangong University, Tianjin 300387, China; 2313910131@tiangong.edu.cn (S.D.); 2431131387@tiangong.edu.cn (J.S.); 2431131406@tiangong.edu.cn (Y.Z.); 2531131493@tiangong.edu.cn (Y.G.); 2531131490@tiangong.edu.cn (S.C.)

**Keywords:** hippocampus, synaptic plasticity, magnetic stimulation, ELF-MF, repetitive transcranial magnetic stimulation, micromagnetic stimulation

## Abstract

**Highlights:**

**What are the main findings?**

- Magnetic stimulation regulates hippocampal synaptic plasticity in a frequency- and state-dependent manner: ELF-MF commonly exerts bidirectional effects with developmental sensitivity, whereas HF-rTMS more consistently restores impaired LTP and related cognitive function under pathological conditions.
- The effects of cross-frequency magnetic stimulation converge on a multilevel mechanistic framework involving Ca^2+^ dynamics, BDNF/TrkB-associated neurotrophic and structural remodeling, and system-level adaptation; micromagnetic stimulation extends this modulation to focal, subregional control of the hippocampus.

**What are the implications of the main findings?**

- The biological effects of hippocampal magnetic stimulation should be interpreted within the combined context of stimulation parameters and network state, rather than on the basis of frequency alone.
- Standardized parameter mapping, biomarker-guided stratification, and miniaturized or multisite stimulation platforms are needed to support the development of individualized and closed-loop precision neuromodulation strategies.

**Abstract:**

Magnetic stimulation modulates hippocampal synaptic plasticity in a parameter-dependent manner with effects shaped by stimulation frequency, intensity, waveform, and exposure conditions. Low-intensity magnetic fields generate induced electric fields that can influence neuronal membrane excitability and neural network activity. Hippocampal long-term potentiation (LTP) and long-term depression (LTD) are widely used as important readouts for evaluating the neurobiological effects of magnetic stimulation. Previous studies have shown that extremely low-frequency magnetic fields (ELF-MFs) may enhance, inhibit, or have no effect on LTP. These divergent effects appear to depend on stimulation conditions, developmental stage, and the baseline state of the neural network. High-frequency repetitive transcranial magnetic stimulation (HF-rTMS) has been reported to promote the recovery of LTP-like plasticity, improve synaptic structure, and regulate the expression of neurotrophic factors in some aging or pathological models. However, its effects are still influenced by both stimulation parameters and biological states. The underlying mechanisms may involve multiple levels of regulation, including Ca^2+^ dynamics, neurotrophic signals, glutamate receptor dynamics, mitochondrial function, and network oscillations. However, these mechanisms have been inferred mainly from various experimental models, and their interactions, temporal sequence, and causal relationships still need further clarification. Micro-magnetic stimulation (μMS) offers a potential technical approach for improving the spatial selectivity of local regulation in the hippocampus. Arrayed and wireless μMS platforms have further expanded their potential applications. However, available evidence has been obtained primarily from in vitro experiments and animal models. This review summarizes the effects of magnetic stimulation with different parameter configurations on hippocampal synaptic plasticity, integrates the biological mechanisms underlying cross-frequency magnetic neuromodulation, and discusses current challenges in the field of magnetic neuromodulation and future directions toward translational development.

## 1. Introduction

Synaptic plasticity provides a fundamental biological basis for adaptation, information storage, and memory formation in the central nervous system. long-term potentiation (LTP) and long-term depression (LTD) jointly maintain the dynamic balance of neural networks. The hippocampus is a key brain region involved in cognition, memory, and emotional processing, and its Schaffer collateral–CA1 pathway, recurrent CA3 circuitry, and perforant path–dentate gyrus pathway are classic models for studies of plasticity regulation [1,2]. Impaired hippocampal LTP has been reported in several neuropsychiatric and neurological disorders, including Alzheimer’s disease, depression, and epilepsy, and is often accompanied by cognitive deficits and affective disturbances [3,4,5]. Accordingly, the restoration or reshaping of hippocampal network function by exogenous physical stimulation has become an important direction in neuromodulation research.

The hippocampus is particularly suitable for studies of magnetic neuromodulation because of its central role in learning and memory, well-organized circuit architecture, and established paradigms for assessing synaptic plasticity. Classical hippocampal pathways, including the perforant path-dentate gyrus and Schaffer collateral–CA1 pathway, have long been used to investigate LTP and activity-dependent synaptic plasticity [6]. Its role in adult neuroplasticity, aging, and neurological disorders further supports its relevance as a potential experimental and translational target for magnetic neuromodulation [7,8,9].

Low-intensity magnetic fields generate induced electric fields that can influence neuronal membrane polarization without directly eliciting action potentials in resting neurons and are therefore generally regarded as a form of subthreshold physical stimulation [10,11]. Even under subthreshold conditions, magnetic stimulation can modulate hippocampal neuronal output and alter the excitability of the nervous system. Such approaches have been explored experimentally, and in some settings, clinically in disorders such as depression, Alzheimer’s disease, and epilepsy. In hippocampus-related studies, changes in LTP and LTD are widely used as electrophysiological readouts of the neurobiological effects of magnetic stimulation.

Current evidence indicates that the effects of magnetic stimulation on hippocampal synaptic plasticity vary substantially across experimental conditions. Previous studies suggest that these divergent outcomes may arise from the interplay between stimulation parameters and biological context. Across experimental systems, magnetic stimulation has been associated with enhanced or reduced synaptic transmission, or no detectable change; however, the factors determining the direction and magnitude of these responses remain incompletely understood [12,13,14]. Therefore, clarifying how stimulation parameters interact with biological state to shape synaptic plasticity represents a key challenge in magnetic neuromodulation research.

In pathological models, magnetic stimulation-mediated regulation of hippocampal synaptic plasticity appears to be more context-dependent. Studies in aging, vascular dementia, and epilepsy models have reported changes in cognition-related behavior, synaptic plasticity indices, and abnormal network activity after magnetic stimulation [15,16,17,18]. However, these effects are influenced by both stimulation parameters and disease context. Taken together, current evidence suggests that magnetic stimulation does not appear to regulate neural plasticity through a single stimulation parameter or mechanism. Instead, its effects arise from interactions among physical stimulation parameters, model state, and biological context.

Accordingly, this review summarizes recent progress in magnetic modulation of hippocampal synaptic plasticity across frequency-dependent magnetic stimulation paradigms, with an emphasis on extremely low-frequency magnetic fields (ELF-MFs), high-frequency repetitive transcranial magnetic stimulation (HF-rTMS), and emerging micro-magnetic stimulation (μMS). It further summarizes current evidence regarding potential mechanisms spanning cellular and molecular signaling, synaptic structural remodeling, and system-level adaptation. The review also compares the characteristics and limitations of different magnetic stimulation paradigms, and discusses current technical limitations, safety considerations, and future directions for precision magnetic neuromodulation.

## 2. Differential Regulation of Hippocampal Synaptic Plasticity Across Frequency-Related Magnetic Stimulation Paradigms

Considering the combined influence of stimulation parameters and biological context, this section compares the effects of ELF-MF, HF-rTMS, and μMS on hippocampal synaptic plasticity across experimental models, focusing on phenomenological outcomes including LTP enhancement, LTD inhibition, regulation of abnormal neural activity, and changes in cognition-related behavior. In contrast to the mechanistic analysis presented in Section 3, this section summarizes observed response patterns at the phenomenological level and relates them to the corresponding stimulation conditions and biological states.

### 2.1. Parameter-Dependent Regulatory Effects of ELF-MF

The direction of ELF-MF-related modulation of hippocampal synaptic plasticity varies across experimental conditions. In ex vivo hippocampal slices following LTP induction, several studies have reported that ELF-MF exposure enhances LTP expression and suppresses LTD responses. Xia et al. showed that 15 Hz, 2 mT ELF-MF reduced LTP in the Schaffer collateral–CA1 pathway and promoted an LTD-like process [1]. This effect was dependent on the Ca^2+^/calcineurin signaling pathway. Further studies by Zheng et al. demonstrated that waveform also influences the direction and magnitude of modulation: under the same frequency and intensity conditions, continuous sinusoidal stimulation produced stronger inhibition than pulsed stimulation containing harmonic components, and the inhibitory effect increased with longer exposure duration [12]. Beyond frequency, intensity, and waveform, the temporal structure of the stimulation signal may also influence plasticity responses to magnetic stimulation. Dong et al. derived time-varying magnetic fields (2 mT) from different musical rhythm frequencies and found that the high-frequency rhythm-derived magnetic field increased LTP amplitude by 41.8%, indicating a larger effect than the medium- and low-frequency groups [13]. These findings suggest that temporal signal organization may influence hippocampal plasticity beyond conventional frequency and intensity parameters, although the underlying mechanism and reproducibility require further investigation.

However, ELF-MF exposure does not produce uniformly inhibitory effects. Prolonged low-intensity exposure (50 Hz, 100 μT) enhanced LTP and synaptic transmission efficiency in the perforant path-dentate gyrus pathway [15]. Under conditions of abnormal excitability, the direction of modulation may shift further. In epilepsy models, low-intensity magnetic stimulation reduced discharge amplitude, whereas high-intensity, long-duration stimulation blocked seizure progression [19,20]. In an Alzheimer’s disease model, the effects of 40 Hz, 10 mT magnetic field exposure also varied with age and exposure pattern [21].

In addition to LTP enhancement or LTD inhibition, some studies have reported limited beneficial effects or potential adverse biological responses. For instance, Li et al. [22] found that ELF-MF altered AMPAR/NMDAR subunit expression in the hippocampus, entorhinal cortex, and prefrontal cortex, but did not improve spatial learning and memory in rats. Liu et al. [23] reported that chronic ELF-MF stimulation induced anxiety-like behavior, whereas Manikonda et al. [24] found that ELF-MF exposure was associated with oxidative stress in brain tissue. These findings indicate that ELF-MF does not produce a consistent direction of plasticity modulation. Its biological effects should be interpreted in relation to stimulation parameters, biological state, and outcome measures.

Taken together, these studies suggest that the effects of ELF-MF on hippocampal synaptic plasticity are highly condition-dependent. Reported outcomes include LTP enhancement, LTP inhibition, modulation of abnormal neural activity, and limited or absent behavioral effects. Current evidence has not yet established a unified response pattern. Further studies using standardized experimental designs and systematic cross-model comparisons are needed to clarify the factors contributing to the divergent effects of ELF-MF across experimental systems.

### 2.2. Developmental Stage-Dependent Effects of ELF-MF on Hippocampal Plasticity

The effects of ELF-MF on hippocampal synaptic plasticity vary across developmental stages. Zhao et al. found that the inhibitory effect of 15 Hz, 2 mT ELF-MF on LTP followed a clear age gradient, being strongest at postnatal day 8 (P8), weaker at P15, further attenuated at P22, and absent at P29 [14]. Consistent with this finding, studies in rat brain slices during embryonic and early postnatal periods also showed that ELF-MF was associated with changes in synaptic efficacy in different directions or amplitudes across developmental windows. These results suggest that the maturation state of early neural networks may contribute to differences in plasticity phenotypes after magnetic field exposure [25].

In addition to the developmental stage, neuronal maturity may also influence ELF-MF related plasticity. In primary cultured hippocampal neurons, single or repeated 50 Hz, 2 mT magnetic field stimulation induced detectable biological responses in immature neurons [26]. Studies in the adult hippocampus further indicate that ELF-MF may affect neurogenesis and the survival of newborn neurons [27,28]. Taken together, these findings suggest that ELF-MF-related effects on hippocampal plasticity are influenced by neural maturation. During early development, the response may mainly involve changes in synaptic transmission and LTP levels, whereas in the mature hippocampus, it may also involve neurogenesis-related processes.

### 2.3. HF-rTMS: Plasticity Regulation and Function-Related Effects in Pathological Models

Compared with the heterogeneous effects reported for ELF-MF, HF-rTMS has been more frequently examined in studies assessing plasticity-related regulation and functional outcomes in aging and pathological models [3,4,29,30]. In a natural aging model, Ding et al. used electrophysiological recordings to evaluate the effects of 20 Hz rTMS after 14 days of continuous stimulation. The results showed that rTMS partially reversed age-related alterations in the resting membrane potential, action potential threshold, and burst-firing activity of dentate gyrus (DG) granule cells. Behavioral tests further indicated improved cognition-related performance, and these electrophysiological changes were correlated with behavioral outcomes [31]. Ma et al. reported that 5 Hz and 25 Hz repetitive magnetic stimulation improved spatial learning performance in aged mice, and this improvement was accompanied by structural remodeling of hippocampal synapses and altered levels of plasticity-related molecular markers, including BDNF and phosphorylated CREB (p-CREB) [16]. Hong et al. showed that in a cerebrovascular disease model, 20 Hz rTMS increased synaptic structural parameters and was accompanied by improvements in cognitive-behavioral performance [32]. In addition, studies in vascular dementia models have shown that 5 Hz stimulation can restore LTP in the CA3-CA1 pathway and improve spatial learning ability [17]. Together, these studies demonstrate multilevel responses at electrophysiological, behavioral, structural, and molecular levels. However, changes in LTP should not be directly equated with cognitive improvement, as these outcomes reflect distinct levels of neural function.

It is worth noting that some low-frequency stimulation paradigms or ELF-MF exposure can also produce favorable electrophysiological or behavioral responses under specific pathological conditions. For example, 1 Hz rTMS restored hippocampal LTP in a mouse model of Alzheimer’s disease [33], and 50 Hz ELF-MF increased adult neurogenesis [34]. These findings indicate that magnetic stimulation-induced plasticity is not solely dependent on stimulation frequency alone. Different stimulation paradigms may elicit distinct electrophysiological, molecular, or behavioral responses depending on the experimental context. Moreover, because animal models, stimulation protocols, and outcome measures vary considerably across studies, the observed effects should be interpreted as condition-specific responses rather than generalized evidence of cognitive improvement.

In addition, the effects of HF-rTMS show a clear intensity dependence [35,36]. Chen et al. found that 25 Hz stimulation was associated with enhanced LTP-like plasticity and improved object recognition memory at 100% of the resting motor threshold (RMT). In contrast, no such effects were observed at 60% or 120% intensity, yielding a typical inverted U-shaped response curve [18].

In summary, HF-rTMS has been reported to influence LTP-like plasticity, alter neuronal excitability, and modify synaptic structural indicators in some aging and disease models. In several studies, these changes were accompanied by alterations in function-related behavioral outcomes. However, these effects are not consistent across all models, stimulation frequencies, or stimulation intensities. Therefore, findings from different studies should be interpreted in relation to the experimental model, stimulation protocol, and outcome measures. These results provide a foundation for further mechanistic analysis.

## 3. Multiscale Biological Mechanisms Underlying Cross-Frequency Magnetic Regulation of Hippocampal Synaptic Plasticity

**Figure 1:**
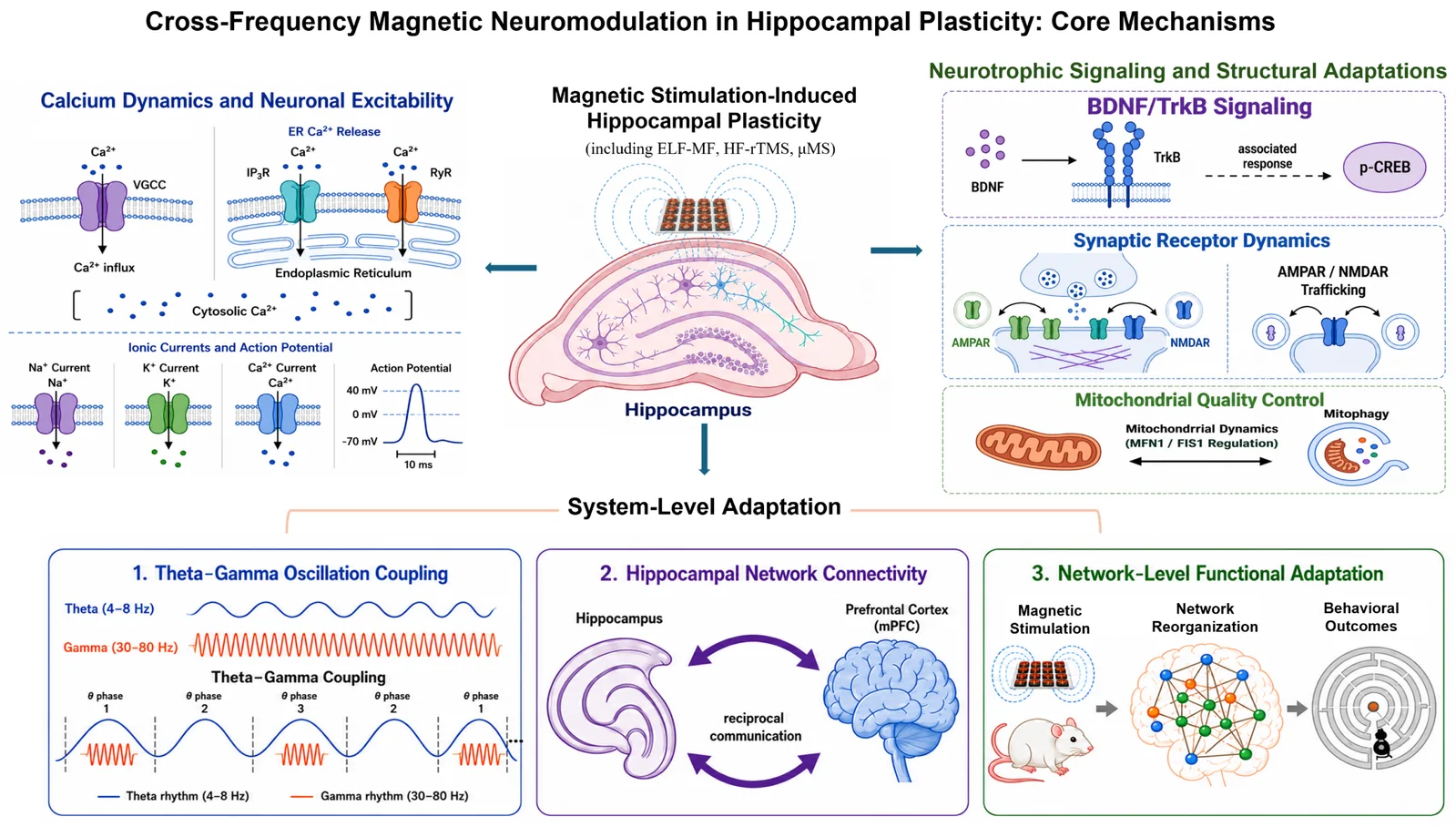
Schematic overview of cross-frequency magnetic neuromodulation in hippocampal plasticity. Magnetic stimulation paradigms including ELF-MF, HF-rTMS, and μMS may regulate hippocampal plasticity through multilevel mechanisms. The left panel depicts calcium dynamics and neuronal excitability, covering VGCC-mediated Ca^2+^ influx, endoplasmic reticulum Ca^2+^ release, ionic currents, and action potential changes. The right panel summarizes molecular and synaptic adaptations, including BDNF/TrkB signaling, AMPAR/NMDAR trafficking, and mitochondrial quality control. The lower panels illustrate system-level adaptations, such as theta–gamma coupling, hippocampal–prefrontal connectivity, network reorganization, and behavior-related outcomes.

The plasticity changes observed under the magnetic stimulation paradigms described in Section 2 may involve multiple interacting regulatory processes. This section summarizes current evidence on the mechanisms by which magnetic stimulation regulates hippocampal plasticity, focusing on Ca^2+^ dynamics, neurotrophic signaling, and synaptic structural remodeling, network oscillations, and system-level adaptation. It should be noted that existing mechanistic evidence is derived mainly from observations across different experimental models and biological scales. Therefore, the framework proposed here should be understood as a multiscale conceptual model based on current evidence, rather than as a linear causal chain verified in a single experimental system [3,37]. Based on this rationale, Figure 1 summarizes the proposed multiscale conceptual framework for cross-frequency magnetic regulation of hippocampal synaptic plasticity.

### 3.1. Calcium Dynamics in Magnetic Stimulation-Mediated Regulation of Hippocampal Plasticity

Changes in intracellular Ca^2+^ concentration are considered an important signaling component underlying magnetic stimulation-induced hippocampal synaptic plasticity. In hippocampal slice experiments, Xia et al. found that exposure to 15 Hz, 2 mT ELF-MF suppressed LTP and enhanced LTD-like responses [1]. This effect was mediated by Ca^2+^ influx and the calcineurin pathway, and it was abolished by the voltage-gated calcium channel blocker CdCl2. In contrast to this extracellular Ca^2+^ influx mechanism, Zhao et al. showed that the inhibitory effect of 15 Hz, 2 mT ELF-MF on LTP in early developmental stages was associated with IP_3_R-mediated release from intracellular calcium stores [14]. Blockade of IP3Rs altered the inhibitory effect across age groups.

The interaction between baseline intracellular Ca^2+^ levels and magnetic field frequency may influence the direction of plasticity regulation. Zheng et al. reported that low intracellular Ca^2+^ levels enhanced the inhibitory effect of a low-frequency magnetic field (15 Hz) on LTP, whereas high Ca^2+^ levels strengthened the facilitatory effect of a high-frequency magnetic field (70 kHz) on LTP [38]. These findings suggest that Ca^2+^ dynamics may serve as an important link between magnetic field parameters and synaptic responses. Differences in Ca^2+^ sources, including extracellular influx and release from intracellular calcium stores, may interact with baseline cellular state to shape the final plasticity outcome.

Notably, magnetic-field-induced changes in Ca^2+^ dynamics are not confined to postsynaptic signaling, but also extend to intrinsic neuronal excitability. Bertagna et al. showed that exposure to 50 Hz, 1 mT ELF-MF for 60 min reduced inward sodium current amplitude and transient outward potassium current amplitude in hippocampal CA1 pyramidal neurons by approximately 37% and 46%, respectively, while persistent outward current remained unchanged [39]. These effects were blocked by pretreatment with the ryanodine receptor inhibitor dantrolene or the SERCA inhibitor cyclopiazonic acid, indicating dependence on intracellular Ca^2+^ dynamics. Tan et al. further demonstrated that repetitive magnetic stimulation also systematically altered the intrinsic excitability of hippocampal CA1 pyramidal neurons as well as the kinetic properties of sodium, potassium, and calcium channels [40]. These results suggest that ion channel regulation may contribute to functional changes in neurons following magnetic stimulation. However, current evidence linking Ca^2+^ signaling to the effects of magnetic stimulation remains largely correlational and comes from diverse experimental systems. Whether Ca^2+^ signaling serves as a common upstream regulatory process across magnetic stimulation paradigms requires further verification.

This frequency-dependent remodeling of Ca^2+^ dynamics and ionic conductances affects not only synaptic plasticity and membrane excitability in mature neurons, but also extends to neural development and differentiation. Bai et al. observed that, in hippocampus-derived neural stem cells, high-intensity electromagnetic fields promoted cell proliferation, whereas low-frequency exposure preferentially promoted neuronal differentiation [41]. These findings suggest that frequency-dependent magnetic stimulation may influence neural development-related processes.

Overall, Ca^2+^ dynamics are considered a candidate mechanism through which magnetic stimulation may affect neural plasticity. These effects may involve the regulation of synaptic transmission, neuronal excitability, and developmental processes. Differences in Ca^2+^ response patterns across models further indicate that magnetic stimulation effects depend on both stimulation conditions and the physiological state.

### 3.2. Neurotrophic Signaling and Structural Plasticity: BDNF/TrkB-Associated Mechanisms

Specific magnetic stimulation patterns may be associated with changes in neurotrophic factor-related signaling and may contribute to the remodeling of synaptic structure and function [11,29,42]. Zhang et al. showed that, in 15-month-old aged mice, 1 Hz rTMS increased the expression of synaptophysin and GAP43 in the hippocampal CA1 and CA3 regions and concomitantly activated the BDNF-TrkB-Fyn signaling pathway, thereby enhancing structural plasticity [43]. Klimiuk et al. demonstrated that a dose-dependent pattern was also observed in this neurotrophic regulation: 1 mT ELF-MF promoted the expression of neuroplasticity-related trophic factors and adaptive responses, whereas 7 mT exposure suppressed this expression pattern and showed an opposite trend [44], suggesting that different magnetic stimulation intensities may correspond to distinct molecular response patterns. In addition to alterations in Ca^2+^ dynamics, BDNF/TrkB-related signaling is also considered to be involved in magnetic stimulation-associated long-term synaptic remodeling. However, the temporal sequence and direct causal relationships among Ca^2+^ signaling, neurotrophic pathways, and structural changes remain insufficiently validated, and current studies have largely revealed the coordinated involvement of these mechanisms in responses to magnetic stimulation. Ma et al. found that, compared with 25 Hz, 5 Hz produced stronger upregulation of BDNF and p-CREB expression as well as greater structural plasticity in the aging hippocampus [16]. Zuo et al. reported that, in a depression model, 15 Hz rTMS was more effective than 25 Hz in preventing neuronal loss and promoting neurogenesis, whereas 25 Hz showed a greater tendency to enhance synaptic plasticity [45]. Zhang et al. also found that restoration of LTP by 5 Hz rTMS in a vascular dementia model depended on selective upregulation of the NR2B subunit [17].

In addition to changes in neurotrophic signals, transcriptional regulation and changes in early neural activity markers are considered to be involved in structural and functional responses associated with magnetic stimulation. Sun et al. showed that, from a transcriptomic perspective, 10 Hz rTMS reversed widespread hippocampal gene expression dysregulation induced by sleep deprivation [46]. Bontempi et al. reported that some electromagnetic exposure paradigms, even in the absence of overt behavioral impairment, also produced region-specific changes in c-Fos expression in the hippocampus and related cortical regions [47]. These studies suggest that transcriptional regulation and changes in early activity markers may be involved in the long-term neural adaptation processes associated with magnetic stimulation. However, the underlying mechanisms and their relationships with other regulatory processes remain to be elucidated further. In addition to transcriptional programs, postsynaptic receptor dynamics and subcellular organelle homeostasis also participate in long-term structural remodeling. A review by Caballero-Villarraso et al. indicated that the neuroprotective effects of rTMS arise from the coordinated involvement of multiple mechanisms, including glutamatergic and GABAergic regulation, attenuation of oxidative stress, improvement in mitochondrial function, suppression of apoptosis, and LTP/LTD-like remodeling [3,4,37,48]. Within this multidimensional framework, Vlachos et al. demonstrated that 10 Hz repetitive magnetic stimulation induced LTP and dendritic spine remodeling through glutamatergic signaling in organotypic hippocampal slice cultures [49]. This process may involve NMDA receptor activation and the postsynaptic clustering of GluA1-containing AMPA receptors.

From the perspective of network regulation, coordinated multi-target stimulation may further enhance structural remodeling. Zheng et al. reported that coordinated stimulation delivered for 1 min to multiple hippocampal subregions (CA1-CA3-DG) with a 1 × 4 array micromagnetic stimulation device at 2 mT and 70 kHz produced a significantly greater increase in LTP induction than stimulation of one or two regions alone, suggesting that multipoint coordination may strengthen synaptic plasticity by integrating network-level responses within local hippocampal circuits [50]. Song et al. further showed that, in a CUMS depression model, 10 Hz rTMS also improved mitochondrial quality control by upregulating the mitochondrial fusion protein MFN1, downregulating the fission protein FIS1, and promoting autophagy, as reflected by an increased LC3 II/I ratio [51]. In addition to mitochondrial function and synaptic receptor regulation, cytoskeletal dynamics may also contribute to magnetic stimulation-associated long-term structural plasticity. Lobyntseva et al. reported that specific ELF-MF magnetic fields (3.9 Hz and 40 Hz) were associated with changes in neuronal microtubule dynamics and Tau–microtubule interactions [52]. These findings suggest that cytoskeletal regulation may contribute to magnetic stimulation-related structural changes, although its specific role remains to be clarified. However, this line of research is currently based mainly on cellular and molecular observations, and the relationship between cytoskeletal changes and synaptic functional alterations requires further investigation.

### 3.3. System-Level Adaptation: Brain Oscillatory Coupling and Neuroendocrine Modulation

Building on the structural and molecular responses described above, changes in local synaptic efficacy may be associated with network-level alterations, including changes in local field potential (LFP) oscillations, interregional functional coupling, and system-level functional measures [4,53]. Guo et al. observed that, in a rat working-memory model, 5 Hz, 10 Hz, and 15 Hz rTMS enhanced theta–gamma oscillatory coupling in the prefrontal cortex, with the greatest increase observed in the 15 Hz group [54]. This enhanced coupling was associated with a reduced number of training days required to achieve the working memory task performance criterion. These findings suggest that network oscillatory measures may serve as functional indicators of magnetic stimulation-related responses. However, they do not establish a direct causal link between local synaptic changes and behavioral improvement.

At the neuroendocrine level, local magnetic intervention has also been associated with systemic regulatory changes. Lu et al. found that magnetic stimulation of the left prelimbic cortex at 10 Hz, mediated by superparamagnetic iron oxide nanoparticles, increased BDNF levels and suppressed overactivation of the hypothalamic–pituitary–adrenal axis [55]. Taken together, magnetic stimulation-related functional changes may involve contributions from synaptic plasticity, network oscillatory regulation, and neuroendocrine responses. However, the temporal relationships and direct causal links among these biological levels remain insufficiently established.

From a system-level perspective, multi-site co-stimulation and miniaturized, high-spatial-resolution modulation platforms offer new approaches for studying functional regulation in complex neural circuits. However, their underlying mechanisms, long-term stability, and translational potential remain to be further validated. Representative stimulation parameters and the major findings related to these core biological mechanisms are summarized in Table 1.

## 4. Enhancing Spatial Selectivity: μMS and Local Circuit Regulation

Conventional transcranial magnetic stimulation (TMS) has limited focality of the induced electric field, which restricts direct and precise modulation of subregions within deep brain structures such as the hippocampus. μMS and related miniaturized magnetic stimulation technologies provide potential approaches to improve spatial selectivity, enable multi-site control, and achieve programmable stimulation. However, their stability, safety, and translational feasibility remain to be further validated.

### 4.1. Enhanced Spatial Selectivity: Micro-Coils for Submillimeter-Scale Precise Neuromodulation

Ye et al. demonstrated that, in a hippocampal slice model of epileptiform discharges, high-frequency micromagnetic stimulation at 200–400 Hz applied to the CA3 region markedly suppressed local abnormal firing [56]. Importantly, this inhibitory effect remained confined to the stimulated area and did not spread aberrantly into the adjacent CA1 region. Given the pronounced subregional specialization and complex circuit organization of the hippocampus, this form of focal intervention provides an experimental approach for studying local regulation of hyperexcitable activity, such as epileptiform discharges.

In addition to high spatial resolution, the physical spatial configuration of μMS also plays a decisive role in defining its regulatory boundaries. Saha et al. showed that, when the CA3 region was stimulated with microcoils of different orientations, only horizontally oriented coils effectively evoked excitatory postsynaptic potentials (EPSPs) in the CA1 region, whereas vertically oriented coils had little effect [57]. In the same study, high-frequency stimulation significantly reduced the current threshold required to elicit action potentials. These findings suggest that μMS responses may exhibit spatial confinement, orientation dependence, and distance sensitivity. Therefore, coil configuration and target distance may be important factors influencing local stimulation efficacy.

### 4.2. Technological Integration: Targeted Enhancement Through Microarrays, Wireless Communication, and Nanomaterials

Arrayed and wireless platforms further expand the potential of μMS for multi-region and multi-site circuit modulation. Because hippocampal subregions are interconnected through complex anatomical and functional pathways, single-site stimulation may not fully capture coordinated responses across distributed hippocampal circuits. To address this limitation, Zheng et al. developed a 1 × 4 array-based μMS device and applied spatially coordinated intervention to isolated hippocampal tissue at 70 kHz and 2 mT [50]. The results showed that sensitivity to magnetic stimulation differed across subregions, with the strongest response observed in CA1, where LTP increased by approximately 14.67%. More importantly, coordinated stimulation of multiple targets (CA1-CA3-DG) produced a significantly greater enhancement of LTP than stimulation of one or two subregions alone. From both engineering and circuit perspectives, spatially distributed stimulation may offer a strategy to investigate how different hippocampal subregions and their combinations contribute to synaptic plasticity. In a related direction, Dong et al. extended precise regulation of the Schaffer collateral–CA1 pathway into an in vivo framework involving awake, freely moving animals through a millimeter-scale wireless neural stimulator, thereby further overcoming the physical constraints of conventional transcranial coils and enabling long-term in vivo neuromodulation studies [58].

Beyond arrayed and wireless approaches, material-assisted strategies have also been explored to improve the efficiency of energy transfer between magnetic stimulation systems and target tissues. Lu et al. enhanced local tissue magnetic responsiveness to an external magnetic field by injecting superparamagnetic iron oxide (SPIO) nanoparticles into the target area [55]. This approach may reduce stimulation energy requirements and improve local magnetic modulation efficiency. However, material-assisted strategies remain at an exploratory stage, and their long-term biological safety, biodistribution, and effects on neural function require further investigation.

In conclusion, arrayed and wireless μMS platforms have expanded the experimental capabilities of local magnetic stimulation, while nanomaterial-assisted enhancement offers a potential route to improve coupling between external fields and target tissue. These advances provide an engineering basis for parameter optimization and local circuit regulation. However, their long-term stability, biological safety, and feasibility for higher-level neural applications remain to be further verified.

## 5. Current Challenges and Future Perspectives

**Figure 2:**
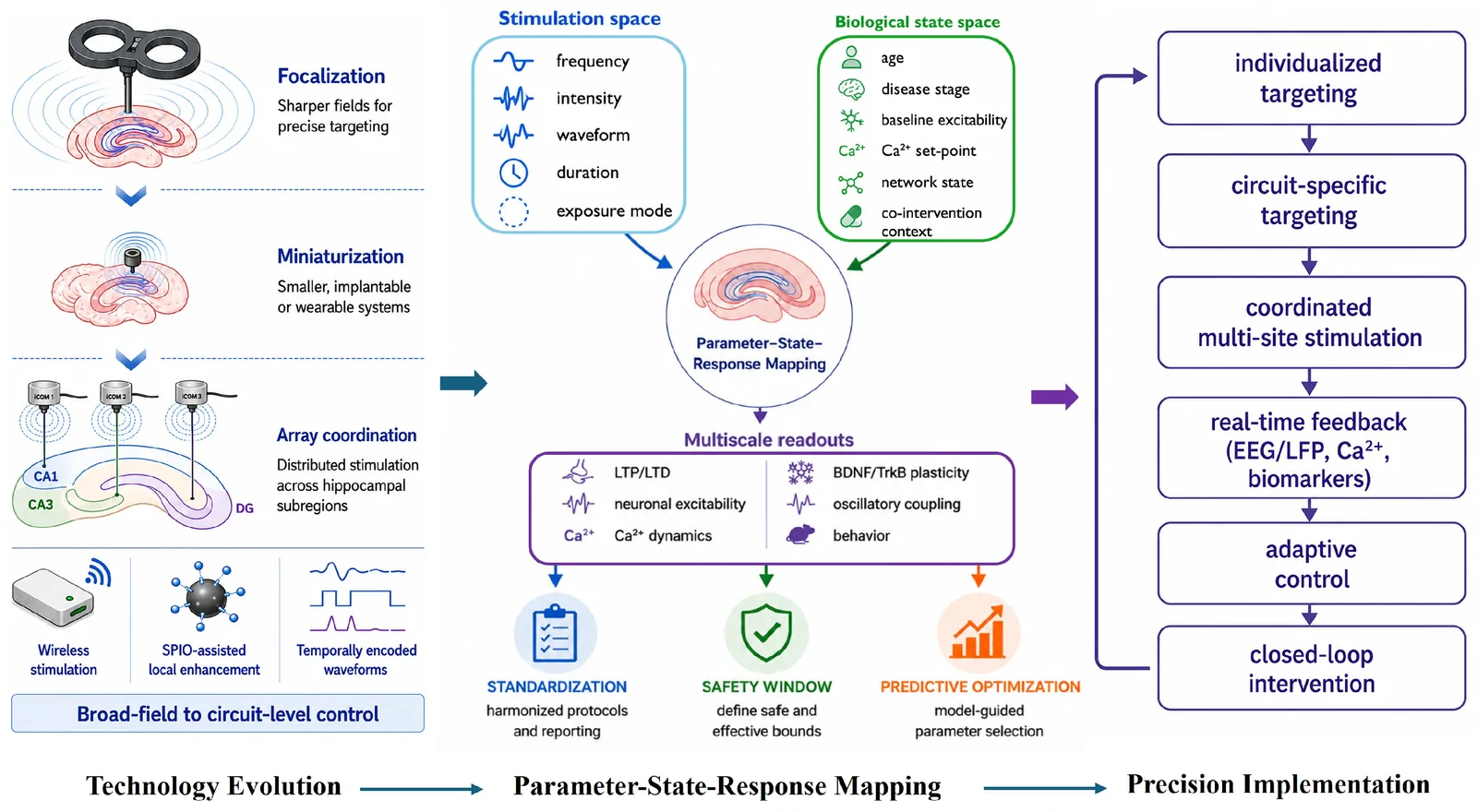
Roadmap for the development of precision hippocampal magnetic stimulation. Technological advances from focalization and miniaturization to array coordination enable a transition from broad field stimulation toward circuit-level control. Integrating stimulation parameters, biological state variables, and multiscale readouts supports standardized protocols, safety-window definition, predictive optimization, and ultimately individualized, feedback-guided closed-loop interventions.

Building on the discussions of hippocampal synaptic plasticity, potential mechanisms, and advances in μMS technology presented in previous sections, this section discusses key challenges for research standardization and clinical translation. Existing evidence suggests that variability in magnetic stimulation outcomes cannot be explained by stimulation frequency alone. Instead, it is also related to stimulation dose, waveform, induced electric field distribution, developmental stage, disease model, baseline network activity, and outcome measures [59,60]. Accordingly, this section considers multidimensional sources of variability, safety boundaries, challenges in translating human rTMS findings toward precise hippocampal modulation, and future directions for individualized closed-loop neuromodulation. Figure 2 illustrates a conceptual roadmap, including technology evolution, parameter–state–response mapping, and precision implementation in hippocampal magnetic stimulation.

### 5.1. Multidimensional Sources of Variability in Magnetic Stimulation Effects: Parameter Combinations, Field Characteristics, and Model State

Current evidence indicates that magnetic stimulation can enhance LTP, inhibit LTD, or produce no detectable change in hippocampal plasticity. These different outcomes should not be interpreted as direct contradictions between studies. Rather, they suggest that magnetic stimulation effects are influenced by interactions among multiple experimental factors. Frequency is only one dimension of these influencing factors. Stimulation intensity, duration, total pulse number or total dose, interstimulation interval, repeated stimulation schedule, and target location can all affect the actual stimulation exposure experienced by the tissue [35,36,61]. Thus, even when the nominal stimulation frequency is the same, different parameter combinations may produce different patterns and magnitudes of synaptic plasticity changes [59,62].

Interactions among stimulation factors are also an important source of divergent findings. Existing ELF-MF studies indicate that stimulation intensity, exposure pattern, and biological state do not act independently, but may interact to influence neural responses to magnetic stimulation. For example, different intensities and repeated exposure schedules can alter molecular response patterns, whereas the same nominal parameter set may produce different plasticity outcomes across age stages or disease states [21,44]. In addition, developmental changes in synaptic maturation, Ca^2+^ dynamics, and network excitability thresholds may modify hippocampal responses to magnetic stimulation [25]. Therefore, comparisons based on a single parameter may be insufficient to explain differences across studies when parameter combinations and model states are not considered.

Stimulation waveform and local field distribution characteristics are additional sources of variability. Different waveforms have distinct temporal structures and rates of current change, which may influence membrane potential dynamics, neuronal excitability thresholds, and plasticity responses after repeated stimulation. Coil structure, pulse direction, stimulation angle, and target anatomy further shape the spatial distribution and effective range of the induced field. Current TMS studies have shown that pulse waveform and directionality can alter cortical excitability aftereffects, while coil design and gyral geometry also influence the actual electric field distribution [63,64,65,66,67]. Therefore, studies reporting similar frequencies or output intensities may still deliver different stimulation distributions and effective stimulation regions to target tissues.

Biological state further influences the range of responses to magnetic stimulation. Developmental stage, aging, disease severity, baseline excitability, synaptic plasticity reserve, and abnormal network activity may all influence whether a given protocol enhances, inhibits, or fails to alter plasticity. In pathological models, magnetic stimulation may modulate impaired plasticity, suppress abnormal excitatory activity, or further disturb an already vulnerable stress state. These outcomes depend on the physiological or pathological state of the model. Thus, “state dependence” should be analyzed in relation to disease stage, baseline network state, stimulation dose, and target field range, rather than treated only as a general conceptual explanation [68,69,70].

In conclusion, discrepant findings in the literature are better understood as the combined result of multiple experimental factors. Stimulation parameters shape the effective stimulation dose, waveform and coil structure influence local electric field distribution, developmental or disease state influence network response thresholds, and outcome measures capture different levels of response to the same stimulation paradigm. Future studies should improve comparability by reporting waveform, stimulation duration, interval structure, coil or field-source configuration, target localization, model stage, and baseline state, and by incorporating these variables into outcome analysis.

### 5.2. Safety Boundary: From ELF-MF to Precise Magnetic Neural Stimulation

The safety assessment of ELF-MF cannot be assessed solely based on a single field strength or frequency parameter. Instead, it requires comprehensive consideration of the stimulation dose, stimulation duration, and the physiological condition of the exposed tissue. It is particularly important to note that the biological effects of ELF-MF may be influenced by developmental stage, stress level, and combined intervention factors. Therefore, its safety cannot be simply inferred from findings obtained from adult healthy animal models. Previous studies have shown that some low-intensity or specific chronic exposure paradigms did not produce overt behavioral or histological damage [71,72]. However, under developmental conditions, stress-related states, drug co-exposure, or higher-intensity exposure, ELF-MF may exacerbate anxiety-like behavior, reduce neurotrophic support, or induce stress-related system dysregulation [23,24]. Further studies have shown that prenatal ELF-MF exposure can induce anxiety-related phenotypes [73], whereas combined prenatal stress and ELF-MF exposure may produce synergistic effects through distinct molecular mechanisms [74]. In addition, combined ELF-MF and ketamine exposure was associated with aggravated depression-like behavior and reduced GFAP expression, accompanied by neurodegenerative changes [75]. High-intensity ELF-MF may further increase noradrenergic system sensitivity and elevate anxiety risk [76]. Therefore, the safety range of ELF-MF should be understood as a dynamic window shaped by stimulation parameters, physiological state, and combined intervention factors, rather than as a threshold defined by field intensity alone [77,78].

Apart from ELF-MF, the safety considerations of HF-rTMS further highlights the importance of dose control in repetitive pulse stimulation. Current TMS safety guidelines indicate that rTMS is generally well tolerated when recommended parameters are followed and appropriate subject screening is performed. However, seizures remain a critical adverse event requiring special attention, and seizure risk is related to stimulation intensity, total pulse number, stimulation frequency, and individual baseline state [60]. Compared with low-intensity ELF-MF, HF-rTMS generates stronger local induced electric fields through rapidly changing magnetic fields. Its safety evaluation should therefore consider not only frequency and intensity, but also pulse number, stimulation interval, coil structure, and targeting accuracy. Guidelines published by Lefaucheur et al. and Rossi et al. further emphasize that rTMS should be applied on the basis of clear indications, standardized protocols, strict screening, and long-term follow-up [60,79].

Different magnetic stimulation techniques operate across different spatial and temporal scales and serve different application contexts. Safety findings obtained for one stimulation modality cannot be directly extrapolated to another. For deep brain regions such as the hippocampus, conventional rTMS, deep TMS, and μMS face different challenges, including limited spatial focality, targeting accuracy, and long-term implant safety. Future development of precise magnetic neuromodulation therefore requires safety evaluation systems matched to the technical characteristics of each modality, with attention to acute tolerance, long-term tissue responses, and network-level functional effects [80,81].

### 5.3. Transition from Animal Models to Human Applications: Challenges in Hippocampal Magnetic Regulation

Although this review focuses on mechanisms of magnetic modulation of hippocampal synaptic plasticity, direct evidence for magnetic stimulation-induced changes in hippocampal LTP-like plasticity is mainly derived from rodent models, isolated hippocampal slices, and organotypic culture systems. In human rTMS research, direct measurement of hippocampal synaptic plasticity remains technically limited. Most studies therefore rely on indirect indicators, such as cognitive scales, neuropsychological tests, electroencephalographic activity, functional connectivity, or cortical excitability, to evaluate the modulation of hippocampus-related network functions by rTMS [82,83,84].

Existing clinical studies suggest that rTMS may improve some cognitive scale scores and memory-related behavioral measures in conditions such as mild cognitive impairment and Alzheimer’s disease, with most reported adverse events being relatively mild [69,85,86]. However, studies differ substantially in stimulation targets, frequencies, treatment durations, the use of combined cognitive training, individualized modulation strategies, and follow-up periods [70,87,88,89]. Therefore, current clinical evidence supports a potential modulatory effect of magnetic stimulation on hippocampus-related networks and cognitive function, but it remains insufficient to demonstrate direct and reproducible modulation of synaptic plasticity within the human hippocampus.

Because the hippocampus is a deep brain structure, precise magnetic modulation of hippocampal subregions remains challenging due to limitations in spatial targeting and stimulation verification. The induced electric field generated by conventional transcranial coils must pass through the scalp, skull, cerebrospinal fluid, and cortical tissue before reaching deeper structures. Its spatial distribution is influenced by individual anatomy, coil configuration, and stimulation positioning, making direct, precise, and repeatable modulation of hippocampal subregions difficult [59,60]. Improvements in memory and cognition after human magnetic stimulation more likely reflect indirect modulation of corticohippocampal circuits, including frontal, parietal, and default mode network pathways, rather than direct stimulation of the hippocampus itself. Clinical findings should therefore be interpreted together with basic mechanistic evidence, and animal findings on hippocampal LTP changes or behavioral improvement should not be directly extrapolated to direct regulation of the human hippocampus [90,91].

### 5.4. Standardization and Personalization for Precise Magnetic Neuromodulation

The development of individualized magnetic neuromodulation remains limited by inconsistent parameter reporting, insufficient cross-model validation, and limited long-term safety data. Future studies should first establish standardized, comparable frameworks for parameter reporting, electric- or local-field estimation, model stratification, and long-term follow-up evaluation [92]. These foundations are necessary for the further development and validation of individualized intervention strategies.

In clinical magnetic stimulation research, individualized electric field modeling, neuronavigation, and functional connectivity-guided target selection may help reduce the gap between nominal stimulation settings and the actual intracranial electric field distribution. These approaches may also help clarify the applicability boundaries between cortico-hippocampal network modulation and direct hippocampal regulation [87,90,91]. In preclinical research, multi-scale readouts such as local field potentials, brain oscillations [54], and molecular markers [55] can support stimulation-response monitoring and parameter optimization. However, their relationships with behavioral improvement or clinical benefit still require longitudinal validation.

From a technology-development perspective, μMS, arrayed stimulation, wireless platforms, and material-assisted strategies provide potential engineering foundations for improving spatial selectivity and parameter programmability [50,57,58]. A promising future direction is to integrate standardized parameter reporting, individualized electric or local field modeling, multi-scale biomarker monitoring, safety assessment, and closed-loop parameter adjustment into a progressive research framework. If long-term stability, tissue safety, and cross-model reproducibility are verified, hippocampus magnetic neuromodulation may gradually develop from a tool for mechanistic exploration into a neuromodulation strategy with translational potential.

## 6. Conclusions

The effects of magnetic stimulation on hippocampal synaptic plasticity are not determined by a single stimulation frequency or a single mechanism. Instead, they are shaped by stimulation parameters, the spatial and temporal characteristics of stimulation, and biological state. Current evidence shows that different magnetic stimulation paradigms can produce plasticity enhancement, inhibition, or limited responses under different experimental conditions. These findings suggest that magnetic stimulation is better understood as a condition-dependent modulatory process rather than as a fixed plasticity-promoting intervention.

Mechanistically, cross-frequency magnetic regulation may involve coordinated responses across cellular, synaptic, and network levels. Ca^2+^ dynamics, neurotrophic signaling, synaptic structural remodeling, cellular metabolic regulation, and network oscillatory regulation may all contribute to neural adaptation after magnetic stimulation. However, current mechanistic evidence is mainly derived from different experimental models, and a unified causal framework has not yet been established. Future studies should combine multi-scale measurement approaches to clarify how molecular events, synaptic changes, and system-level functional responses are related under different stimulation conditions.

In recent years, μMS and related highly localized magnetic stimulation technologies have provided new approaches for addressing the limited spatial selectivity of conventional magnetic stimulation. Microcoils, arrayed stimulation, wireless power supply, and closed-loop feedback may enable more quantitative and individualized regulation of specific neural circuits. However, these technologies remain at the stage of basic validation, and their long-term stability, safety, and translational feasibility for deep human brain applications require further investigation.

Overall, future hippocampal magnetic neuromodulation should move beyond simple optimization of stimulation parameters toward an integrated framework that integrates standardized parameter systems, individualized field modeling, multi-scale biomarker monitoring, and closed-loop control. With cross-model validation and long-term safety evaluation, magnetic stimulation may progress from a tool for studying neural plasticity mechanisms toward a technical platform for more precise neuromodulation. 

## Figures and Tables

**Table 1:** Representative stimulation parameters and major findings from experimental studies on the core biological mechanisms of cross-frequency magnetic neuromodulation.

Reference	Experimental Subject/Model	Frequency	Field Strength	Stimulating Period	In Vivo/In Vitro	Technology	Exposure Duration/Protocol	Main Findings
Xia et al. (2021) [1]	Rat hippocampal slices; SC-CA1 pathway (animals)	15 Hz	2 mT	10 min	No	ELF-MF	Exposure 10 min after LTP stabilization	LTP was suppressed and LTD was enhanced; the effect was mediated by Ca^2+^ influx and the calcineurin pathway.
Zhao et al. (2023) [14]	Hippocampal slices from rats of different postnatal ages (animals)	15 Hz	2 mT	10 min	No	ELF-MF	After LTP induction; duration NR	Developmental-stage-dependent LTP suppression was associated with IP_3_R-mediated intracellular Ca^2+^ release.
Zheng et al. (2024) [38]	Rat hippocampal CA1 slices (animals)	15 Hz versus 70 kHz	2 mT	3 min	No	EMF	Duration NR	Low baseline Ca^2+^ enhanced low-frequency inhibition of LTP, whereas high baseline Ca^2+^ enhanced high-frequency facilitation of LTP.
Bertagna et al. (2025) [39]	Coronal mouse hippocampal slices; CA1 pyramidal neurons (animals)	50 Hz	1 mT	1 h	No	ELF-MF	60 min exposure	Inward Na^+^ current and transient outward K^+^ current decreased; the effect disappeared after blockade of ryanodine receptors or the sarco/endoplasmic reticulum Ca^2+^-ATPase.
Tan et al. (2013) [40]	Rat hippocampal CA1 pyramidal neurons (animals)	1 Hz	NR	Approximately 400 effective stimulations per day	Yes	LF-rTMS	400 stimuli/day for 7 or 14 days	Increased intrinsic excitability and spontaneous excitatory postsynaptic currents, and altered Na^+^/K^+^/Ca^2+^ channel kinetics.
Bai et al. (2021) [41]	In vitro rat hippocampal neural stem cells (animals)	50 Hz	5 mT (LF); 2.5 T peak induced field/40% output (HF)	LF: 30 min; HF: 10 min	No	LF-EMF/HF-EMF	LF: 30 min/day; HF: 10 min/day	Both paradigms promoted proliferation, whereas low frequency more strongly promoted neuronal differentiation.
Zhang et al. (2015, aging brain) [43]	Aged mouse hippocampus (animals)	1 Hz	110% rMT	Approximately 400 effective stimulations per day	Yes	LF-TMS	14 days	Upregulated SYN, GAP43, BDNF, TrkB, and Fyn, supporting BDNF/TrkB-mediated structural plasticity.
Klimiuk et al. (2026) [44]	Adult rat hippocampal transcriptome (animals)	50 Hz	1 mT versus 7 mT	1 h per day	Yes	ELF-MF	7 days per cycle, 1 h/day for 3 cycles, with 3-week intervals	1 mT promoted pro-plasticity gene expression, whereas 7 mT suppressed related factors and disrupted homeostasis.
Ma et al. (2019) [16]	Aged mice (animals)	5 Hz versus 25 Hz	NR	5 Hz: 20 s per sequence; 25 Hz: 4 s per sequence	Yes	HF-rTMS	1000 pulses/day for 14 days	5 Hz produced stronger upregulation of hippocampal BDNF/p-CREB and greater structural plasticity than 25 Hz.
Zuo et al. (2020) [45]	Mouse model of CUMS-induced depression (animals)	15 Hz versus 25 Hz	NR	15 Hz: 17 × 4 s; 25 Hz: 10 × 4 s	Yes	HF-rTMS	4 weeks	15 Hz was better at preventing neuronal loss and promoting neurogenesis, whereas 25 Hz was more favorable for synaptic plasticity.
Zhang et al. (2015, VaD) [17]	Rat model of vascular dementia (animals)	5 Hz	NR	Each sequence lasts for 2 s, with a sequence interval of 2 s.	Yes	HF-rTMS	Repeated stimulation; protocol NR	LTP restoration was associated with selective upregulation of NR2B.
Sun et al. (2026) [46]	Mice after acute sleep deprivation (animals)	10 Hz	NR	About 7 min per time	Yes	HF-rTMS	Daily stimulation during the intervention period; protocol NR	Partially reversed hippocampal transcriptomic dysregulation and improved behavioral performance.
Bontempi et al. (2024) [47]	Head-focally exposed rats; working memory/reference memory tasks (animals)	900 MHz	BASAR 0.5–6 W/kg (acute); 1 and 3.5 W/kg (behavioral task)	Acute: 2 h; behavioral task: 45 min	Yes	RF-EMF	Acute exposure, 2 h; 45 min before each training session	Induced region-specific c-Fos changes, including in the hippocampus, without obvious cognitive deficits.
Vlachos et al. (2012) [49]	Mouse organotypic hippocampal slice cultures (animals)	10 Hz	NR	9 sequences × 10 s, approximately 5.5 min (including intervals)	No	rMS	Acute repetitive magnetic stimulation	Induced LTP-like enhancement and dendritic spine remodeling through NMDA receptor activation and aggregation of GluA1-containing AMPA receptors.
Song et al. (2025) [51]	Mouse model of CUMS (animals)	10 Hz	NR	17 sequences × 4 h per day	Yes	HF-rTMS	1000 pulses/day for 4 weeks	Improved depressive behavior by promoting mitochondrial fusion, accompanied by upregulation of MFN1, downregulation of FIS1, and increased LC3 II/I ratio.
Guo et al. (2023) [54]	Rat working-memory task model; prefrontal cortical local field potential recording (animals)	5/10/15 Hz	100% motor threshold (approximately 22% maximum stimulator output)	Every sequence lasts for 5 s.	Yes	HF-rTMS	600 pulses/day for 14 consecutive days	Enhanced prefrontal theta-gamma coupling; the 15 Hz group showed the greatest increase, which was associated with fewer training days required to complete the task.
Zheng et al. (2024, 1 × 4 μMS array) [50]	Rat hippocampal CA1, CA3, and DG (animals)	70 kHz	2 mT	1 min	No	μMS	Multisite coordinated stimulation	Multisite coordinated stimulation produced a larger increase in LTP than stimulation of one or two sites.
Lu et al. (2020) [55]	CUMS mice; left PrL-targeted magnetic stimulation mediated by superparamagnetic iron oxide nanoparticles (animals)	10 Hz	0.1 T	5 min per time	Yes	SPIO-mediated targeted magnetic stimulation (c-MSS)	Twice a day, for a total of 5 days	Increased BDNF levels and suppressed HPA-axis hyperactivation, rapidly improving depression-like behavior.

BASAR, brain-average specific absorption rate; cMSS, compound magnetic stimulation system; HPA axis, hypothalamic–pituitary–adrenal axis; NR, not reported in the original text or available abstract; PrL, prelimbic cortex; RF-EMF, radiofrequency electromagnetic field; rMS, repetitive magnetic stimulation; SC-CA1, Schaffer collateral-CA1; SPIO, superparamagnetic iron oxide; VaD, vascular dementia. Caballero-Villarraso et al. is a mechanistic review and was not included as a single experimental paradigm in theTable [48].

## Data Availability

No new data were created or analyzed in this study. Data sharing is not applicable to this article because it is a review based on previously published literature.

## Abbreviations

The following abbreviations are used in this manuscript:

AMPAR	*alpha*-amino-3-hydroxy-5-methyl-4-isoxazolepropionic acid receptor
BDNF	brain-derived neurotrophic factor
CUMS	chronic unpredictable mild stress
DG	dentate gyrus
ELF-MF	extremely low-frequency magnetic field
EPSP	excitatory postsynaptic potential
fEPSP	field excitatory postsynaptic potential
GFAP	glial fibrillary acidic protein
GAP43	growth-associated protein 43
GluA1	glutamate receptor 1
HF-EMF	high-frequency electromagnetic field
HF-rTMS	high-frequency repetitive transcranial magnetic stimulation
HPA axis	hypothalamic–pituitary–adrenal axis
IP_3_R	inositol 1,4,5-trisphosphate receptor
LC3	microtubule-associated protein 1 light chain 3
LTD	long-term depression
LTP	long-term potentiation
MFN1	mitofusin 1
NMDAR	N-methyl-D-aspartate receptor
NR2B	N-methyl-D-aspartate receptor subunit 2B
PrL	prelimbic cortex
PSD-95	postsynaptic density protein 95
RMT	resting motor threshold
rTMS	repetitive transcranial magnetic stimulation
SC-CA1	Schaffer collateral-CA1
SERCA	sarco/endoplasmic reticulum Ca^2+^-ATPase
SPIO	superparamagnetic iron oxide
SYN	synaptophysin
TrkB	tropomyosin receptor kinase B
VaD	vascular dementia
VGCC	voltage-gated calcium channel
VEGF	vascular endothelial growth factor
μMS	micro-magnetic stimulation
